# Spatiotemporal Control of Formation of Dynamic Protein Fiber Assemblies via Photophysical Effects of a Focused Laser Beam

**DOI:** 10.1002/advs.75531

**Published:** 2026-05-17

**Authors:** Hiroshi Y. Yoshikawa, Ren Shirata, Takuya Takeshige, Riki Yoshida, Fumika Kiryu, Kei Takano, Shuma Matsumoto, Reiji Kawanami, Natsumi Sawada, Chi‐Shiun Wu, Yang‐Hsin Shih, Hiromasa Niinomi, Takahisa Matsuzaki, Seiichiro Nakabayashi, Tomoaki Matsuura, Teruki Sugiyama, Ryuzo Kawamura

**Affiliations:** ^1^ Graduate School of Engineering The University of Osaka Suita Osaka Japan; ^2^ Department of Chemistry Saitama University Saitama Japan; ^3^ Department of Applied Chemistry National Yang‐Ming Chiao Tung University Hsinchu Taiwan; ^4^ Department of Applied Chemistry National Chiayi University Chiayi Taiwan; ^5^ International Institute for Sustainability with Knotted Chiral Meta Matter Hiroshima University Hiroshima Japan; ^6^ Earth‐Life Science Institute Institute of Science Tokyo Tokyo Japan; ^7^ Division of Materials Science Graduate School of Science and Technology Nara Institute of Science and Technology Ikoma Japan

**Keywords:** cytoskeletal protein, laser trapping, microtubule network, motor protein, photophysical effects

## Abstract

Spatiotemporal control of the formation of highly ordered, protein fiber assemblies via photophysical effects of a focused laser beam is demonstrated. Focused irradiation with a continuous laser beam at an air/solution interface can accumulate tubulin proteins at/around the laser focus, which leads to the formation of highly ordered microtubule assemblies. The assemblies can exhibit various dynamic behaviors such as translational motion, bundling, and cilia‐like beating with motor protein and chemical energy, revealing their biological activities. The protein accumulation with a focused laser beam is attributed to the local increases in concentration and temperature produced through two‐types of photophysical effects, i.e., laser trapping by optical forces and heat generation by photoabsorption, which can fabricate complex microtubule assemblies without specific photochemical reactions and deuterated water solvents (i.e., physiological conditions). We anticipate that this laser method will provide fundamental insights into the structure‐motion relationship of biomolecular assemblies and expand the bioengineering of protein assemblies.

## Graphical abstract

Tightly focused laser irradiation can accumulate tubulin proteins at/around the laser focus, which leads to the formation of highly ordered microtubule assemblies. The assemblies can exhibit various dynamic behaviors such as radial motion, bundling, and flagella‐like rotation with motor protein and chemical energy, highlighting as a unique tool for spatiotemporal control of protein assembly without chemical/biological modifications.

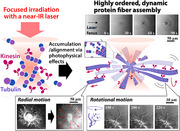

## 1 Introduction

In living cells, dynamic assemblies of protein fibers, called cytoskeletons, play significant roles in various cell functions such as cell division, migration, and adhesion. The fiber assemblies are typically composed of protein filaments (e.g., tubulin and actin) with motor proteins and chemical energy, which exhibit collective and directional motions with dynamic network structures. Thus, understanding the structure‐motion relationship of such a dynamic assembly of protein fibers has been a highly important topic in life sciences. In the biological context, attempts to reconstitute dynamic assemblies in vitro have been made for elucidation of the mechanisms in mitotic spindle formations, aster positionings, and so on, in which microtubules get aligned in radial form or parallel bundles accompanied with specific co‐working proteins [1, 2, 3]. In addition, dynamic assemblies of protein fibers have also attracted much attention in engineering fields for the creation of protein‐based actuators for macrorobotic systems [4, 5, 6, 7, 8, 9, 10, 11, 12]. Owing to such biological and engineering interests, an increasing number of studies have demonstrated reconstruction of dynamic cytoskeletal networks in vitro via self‐organization, which involves macroscopic motions such as muscle‐like contraction [13] and cilia‐like beating [14, 15]. However, the reconstruction method based on self‐organization alone usually lacks the means for spatiotemporally controlling the formation and alignment of fiber assemblies. To solve this problem, some recent studies have used light patterning to trigger the formation of cytoskeletal networks at a chosen location [5, 16]. Here, ultraviolet (UV) or blue light is typically used to activate photochemically active motor proteins, which can crosslink between fibers upon light irradiation. Through the use of the photochemical approach, spatiotemporal control of the formation of active assemblies with cytoskeletal proteins that are capable of macroscopic actuation has been demonstrated. However, the photochemical approach, in principle, requires chemical or genetic modification of molecules (e.g., motor proteins) and light sources with a specific wavelength, which can often limit the degrees of freedom of the experimental system and interactions between molecules without labels.

In this work, we demonstrated spatiotemporal control of the formation of highly ordered, dynamic microtubule assemblies via the photophysical effects of a focused laser beam. Here we utilized increases in concentration and temperature via electric fields and photothermal effects of a focused laser beam, which could promote accumulation and aggregation of tubulin proteins to form microtubules [17]. In particular, the electric field of a focused laser beam is a driving force of laser trapping (i.e., optical tweezers) [18], which has been widely used as a method to manipulate nano/micrometre‐sized particles through an optical force that is as a function of electric field, magnetic flux density, and polarizability of the particles in solution according to Maxwell–Boltzmann theory [19]. In the case of intense focused irradiation of a solution containing molecular solutes, molecular diffusion can be slightly suppressed within the optical potential field, leading to a local increase in the solute concentration [20]. One of the coauthors, Sugiyama, demonstrated for the first time in 2007 that such a local concentration increase induced by laser trapping could be used to control crystallization of an amino acid, glycine [21], and then demonstrated various applications such as crystal polymorph control [20], aggregation‐induced emission of polymers [22], and assembly of protein amyloid fibrils [23]. However, since the focused laser beam with near‐IR wavelength (1064 nm) widely used for laser trapping usually causes a certain amount of heat generation at a laser focus in water (∼20 Kelvin/Watt, K/W) [24], laser trapping of proteins had not been applied to fabrication of dynamic protein assemblies in a water solvent but only to crystallization [25], aggregation [26], and amyloid formation [23, 27] in a deuterated water solvent with less photo‐absorption. In this work, the photophysical (here, electric field and photothermal) effects could increase the local concentration of tubulin and motor protein (kinesin) at the laser focus, which can realize spatial control of the formation of dynamic microtubule fibers in a water solvent without photochemically active crosslinkers (i.e., under conditions more similar to physiological conditions). We also investigated the dynamic motion of the microtubule fiber assemblies in the presence of motor protein (kinesin) and chemical energy and assessed the versatility of the laser‐based technique for the creation of flexible in vitro models of microtubule assemblies that can be used for detailed studies of the structure‐motion relationships of cytoskeletons.

## 2 Results and Discussion

### 2.1 Microtubule Formation by a Focused Laser Beam

**FIGURE 1:**
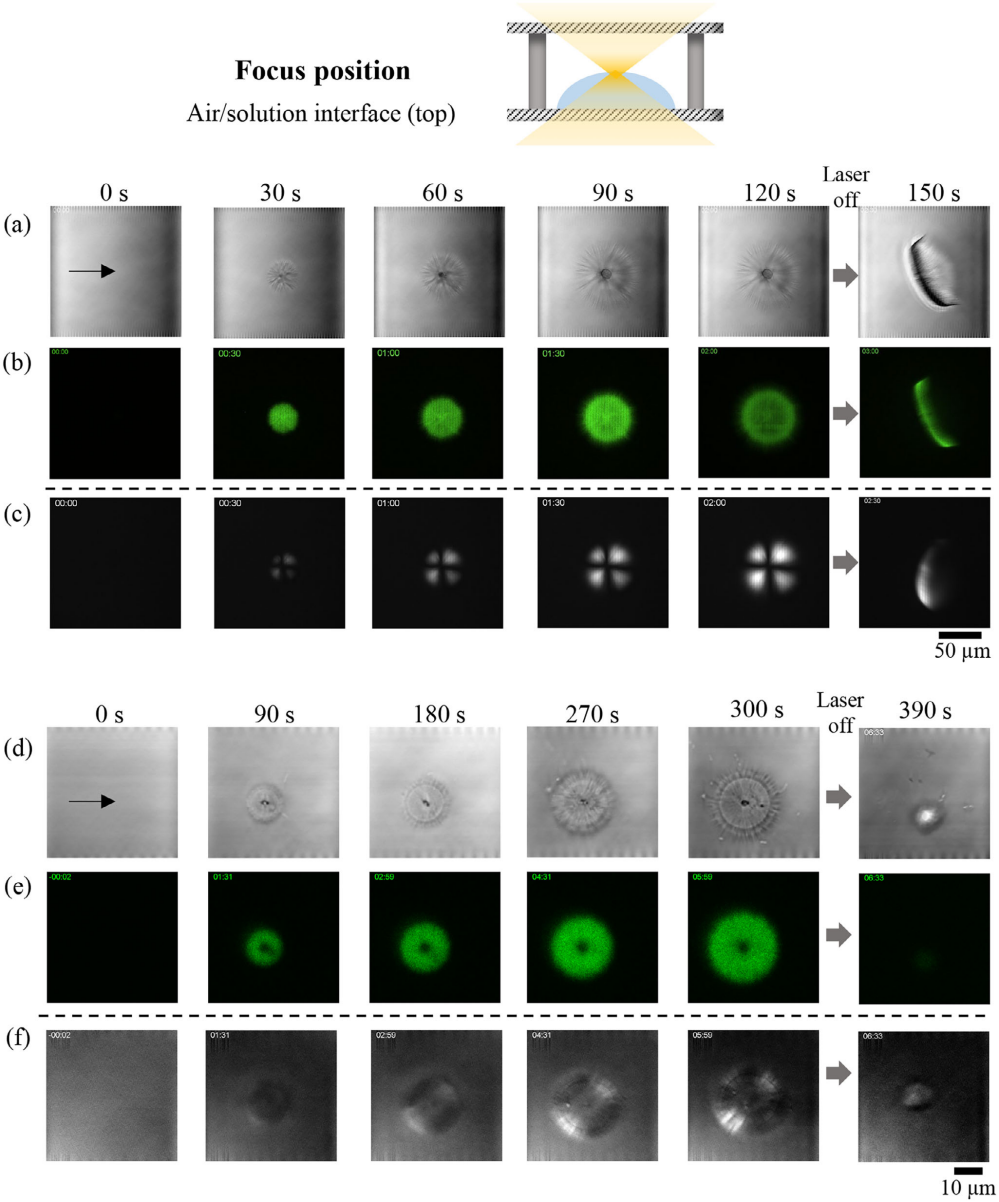
Formation of fiber aggregates by focused laser irradiation at an air/solution interface (top). Laser irradiation with a power of 1.0 W began at *t* = 0 s and stopped at *t* = 120 s for supersaturated tubulin solutions (20 µm, a, b, and c) or stopped at *t* = 300 s for unsaturated tubulin solutions (0.2 µm, d, e, and f). Transmission (a and d), fluorescence (from Alexa 488‐labelled tubulin, b and e), and crossed Nicols (c and f) images were captured. Note that the experiments for crossed Nicols imaging were separately performed from those for fluorescence/transmission imaging because simultaneous imaging was not possible in our experimental setup.

Figure 1 shows the dynamics of tubulin aggregation induced by a focused laser beam at air/solution interfaces. Here, a continuous‐wave laser (1 W) with a wavelength of 1064 nm was focused on the top surface of a hemispherical droplet on a substrate (called the air/solution interface (top)), as typically used in optical trapping‐induced crystallization [20, 21]. When a sample solution with a tubulin concentration of 20 µm, at which the solution is supersaturated at room temperature for microtubule growth but no spontaneous nucleation can occur according to the phase diagram [17], was used, a fiber‐like, circular aggregate immediately formed after the onset of laser irradiation (within seconds) (Figure 1a; Movie S1). The aggregate grew from the laser focus with time, and its size reached over 50 µm at *t* = 120 s, which is significantly larger than the size of the laser focus that can be estimated based on the diffraction limit (1.22*λ/NA ∼ 1.4 µm). After the laser irradiation was stopped at *t* = 120 s, the aggregate was released from the laser focus (looks like a rotation of an umbrella‐shaped object) and began floating into the surrounding area without significant dissociation. The corresponding fluorescence images of Alexa 488‐labelled tubulin revealed a significant increase in the fluorescence intensity during laser irradiation (Figure 1b; Movie S2), indicating that the aggregate shown in Figure 1a was formed by tubulin accumulation at the focal spot. In addition, crossed Nicols imaging of tubulin aggregate formation (Figure 1c; Movie S3) revealed clear and contrastive black and white plaid patterns, indicating that the optically anisotropic fibers were radially aligned around the laser focus, as clearly observed from the corresponding bright field images (Figure 1a). The statistical data showed that the average area of the aggregate monotonically increased during laser irradiation (Figure S1). We also found that the size of the aggregate was increased with laser power, whereas no aggregate was formed with the laser power of 0.1 W (Figure S2), implying that photophysical effects were not high enough to exceed the critical concentration and temperature for fiber formation. Notably, tubulin aggregates could be formed even in an unsaturated solution with a tubulin concentration of 0.2 µm, which is far below the critical concentration for microtubule growth (∼15 µm at 20°C) [17] (Figure 1d; Movie S4). In this case, tubulin aggregates showed slower growth than in the case with 20 µm tubulin and reached ∼30 µm in diameter at *t* = 300 s. The fluorescence (Figure 1e; Movie S5) and crossed Nicols (Figure 1f; Movie S6) images revealed tubulin accumulation and fiber alignment are similar to those in the case of 20 µm, whereas a multilayered shell‐like aggregate tended to be formed in the case of 0.2 µm. Actually, Kudo et al. reported the propagation of trapping laser from the focus to the outside of the formed assembly of colloidal particles leads to the formation of a circular‐shape assembly with horn‐like outer layers [28], which seems to partially resemble the multilayered shell‐like aggregate of this study. In addition, after the laser irradiation was stopped at *t* = 300 s, the aggregate started to dissociate and became smaller with time, indicating that the solution around the laser focus gradualy returned to the unsaturated region (0.2 µm tubulin concentration at 20°C) after the laser irradiation was stopped. The dissolution rate of microtubules in the aggregate for 90 s after laser off was switched off (Figure 1e) was the same order of magnitude as the reported rate of microtubule depolymerization (∼12 µm/min) [29]. In addition, such dissolution behavior after the termination of laser irradiation was also reported for optical trapping‐induced crystallization of amino acids from unsaturated solutions [30].

**FIGURE 2:**
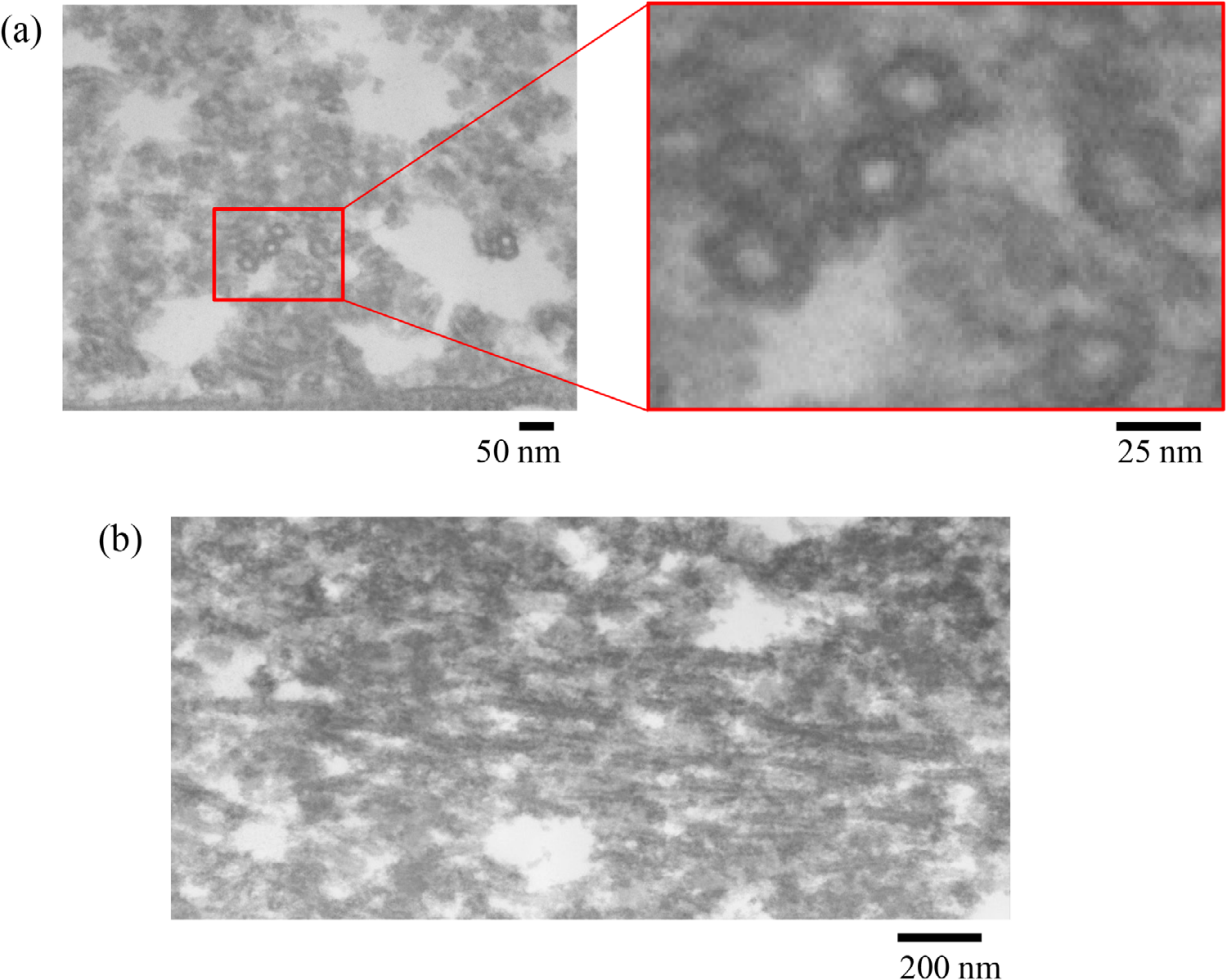
TEM images of a fiber aggregate that formed by focused laser irradiation at an air/solution interface (top). (a) Cross‐sectional image of microtubules. The right image is a magnified view of the red box in the left image. (b) Lengthwise‐sectional image of microtubules.

To clarify whether the aggregates formed by laser irradiation at air/solution interfaces were microtubules or otherwise merely denatured tubulins, the laser‐induced aggregates were extracted from the chamber and their samples embedded in resin were observed with a transmission electron microscope (TEM). The TEM images showed aligned fibers with length ranging from a few hundred nanometres to micrometres (Figure 2). In some parts of the TEM images, ring‐shaped cross sections of the fibers could be found, in which globular proteins were associated to form hollow tubes. The diameter of the tubes was ∼ 25 nm and the apparent number of subunits in the wall approximates 13, which well corresponds to that of the microtubules found most frequently in vivo. These results clearly indicate that focused laser irradiation at the air/solution interface can produce microtubules through the accumulation of tubulins at the laser focus.

### 2.2 Dynamic Motion of Microtubule Assemblies Generated by a Focused Laser Beam

**FIGURE 3:**
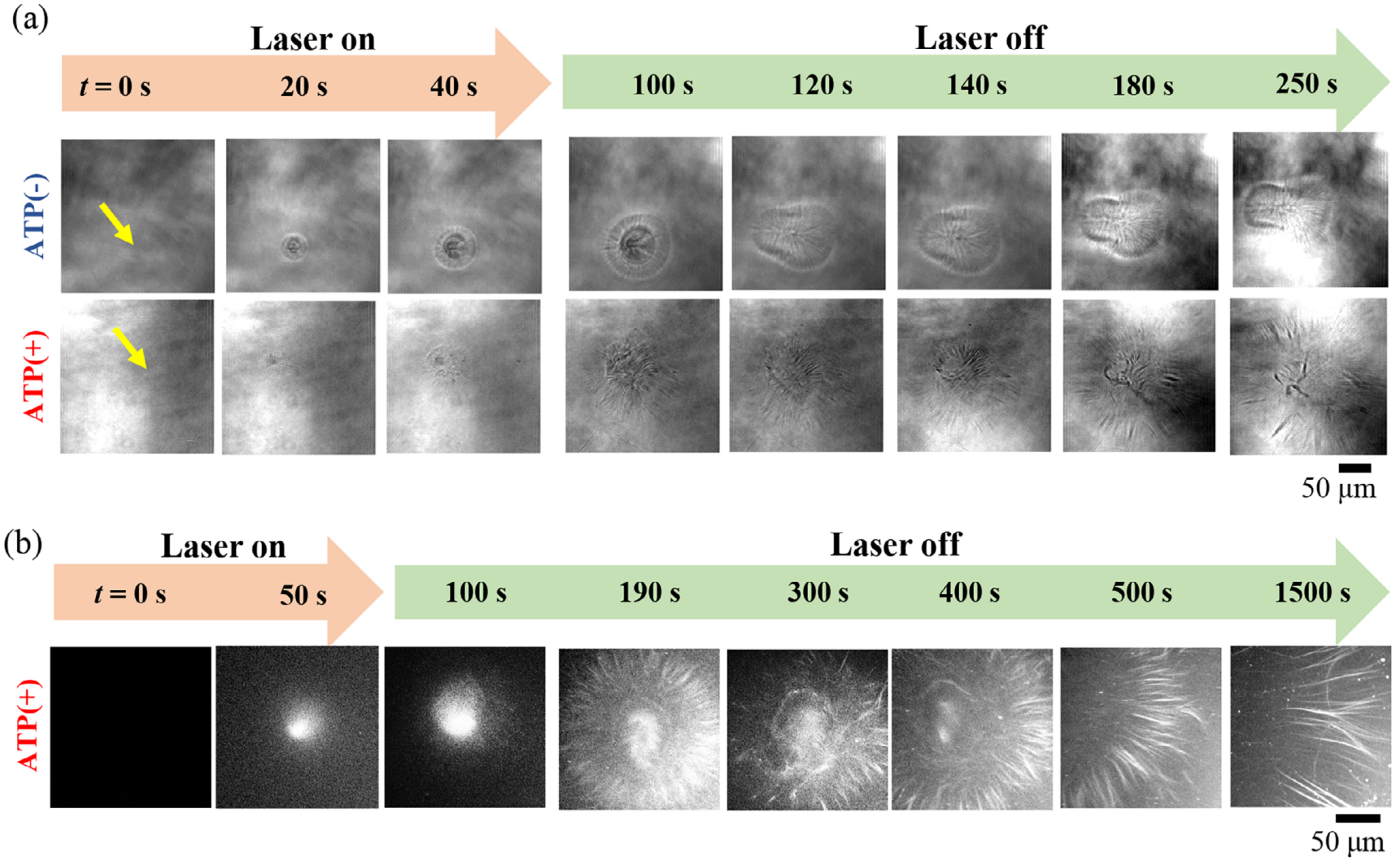
(a) Transmission and (b) fluorescence images of the formation of fiber aggregates via focused irradiation at an air/solution interface (top) in the presence of the motor protein kinesin. The fluorescence signal in (b) is originated from GFP‐fused kinesin. Laser irradiation with a power of (a) 1.0 W or (b) 0.5 W began at *t* = 0 s and stopped at *t* = 100 s. Note that the imaging area in (b) was shifted toward the lower right to track the motion of the fibers (e.g., *t* = 500 and 1500 s). When the laser was switched off, fiber aggregates were mechanically released from the optical forces and exhibited elastic deformation and position shift, which resulted in changes in the imaging focus.

To investigate the biological activity of the highly ordered microtubule assemblies generated by a focused laser beam, we carried out laser experiments with tubulin in the presence of motor protein, kinesin, and/or chemical energy (adenosine triphosphate, ATP). Figure 3a shows a representative result for aggregates made by focused irradiation at an air/solution interface. When a solution containing tubulin and kinesin without ATP was used, focused laser irradiation at air/solution interfaces resulted in the formation of a circular aggregate with fibers (diameter ∼100 µm at *t* = 100 s) (Figure 3a upper), which is consistent with the results obtained with a tubulin solution without kinesin and ATP (Figure 1a). Upon cessation of the laser irradiation, the aggregate was released from the laser focus and slightly deformed (became ellipsoidal shaped) probably due to the elastic response of the aggregates. Afterward, no significant change in the aggregate shape was observed. In contrast, when a solution containing tubulin, kinesin, and ATP was used, the aggregate expanded more rapidly, and its size (diameter ∼150 µm) at *t* = 100 s became approximately 1.5 times larger than that without ATP (Figure 3a lower). Notably, after the laser irradiation was switched off, the aggregate still expanded toward the surroundings, and the diameter increased to ∼300 µm at *t* = 250 s. In addition, some of the fibers formed thicker bundles during expansion. To more clearly visualize the motion of each fiber in the presence of kinesin and ATP, we then carried out fluorescence imaging of aggregate formation by using green fluorescent protein (GFP)‐tagged kinesin (Figure 3b; Movie S7). The fluorescence images showed an increase in the fluorescence intensity of the aggregates, indicating that GFP‐fused kinesin was assembled into the aggregates together with tubulin by focused laser irradiation. Each fiber showed translational‐like motion toward the surroundings even after the laser irradiation was switched off (*t* > 100 s), and the motion still lasted at *t* = 1500 s. In addition, most of the fibers formed longer and thicker bundles with time.

**FIGURE 4:**
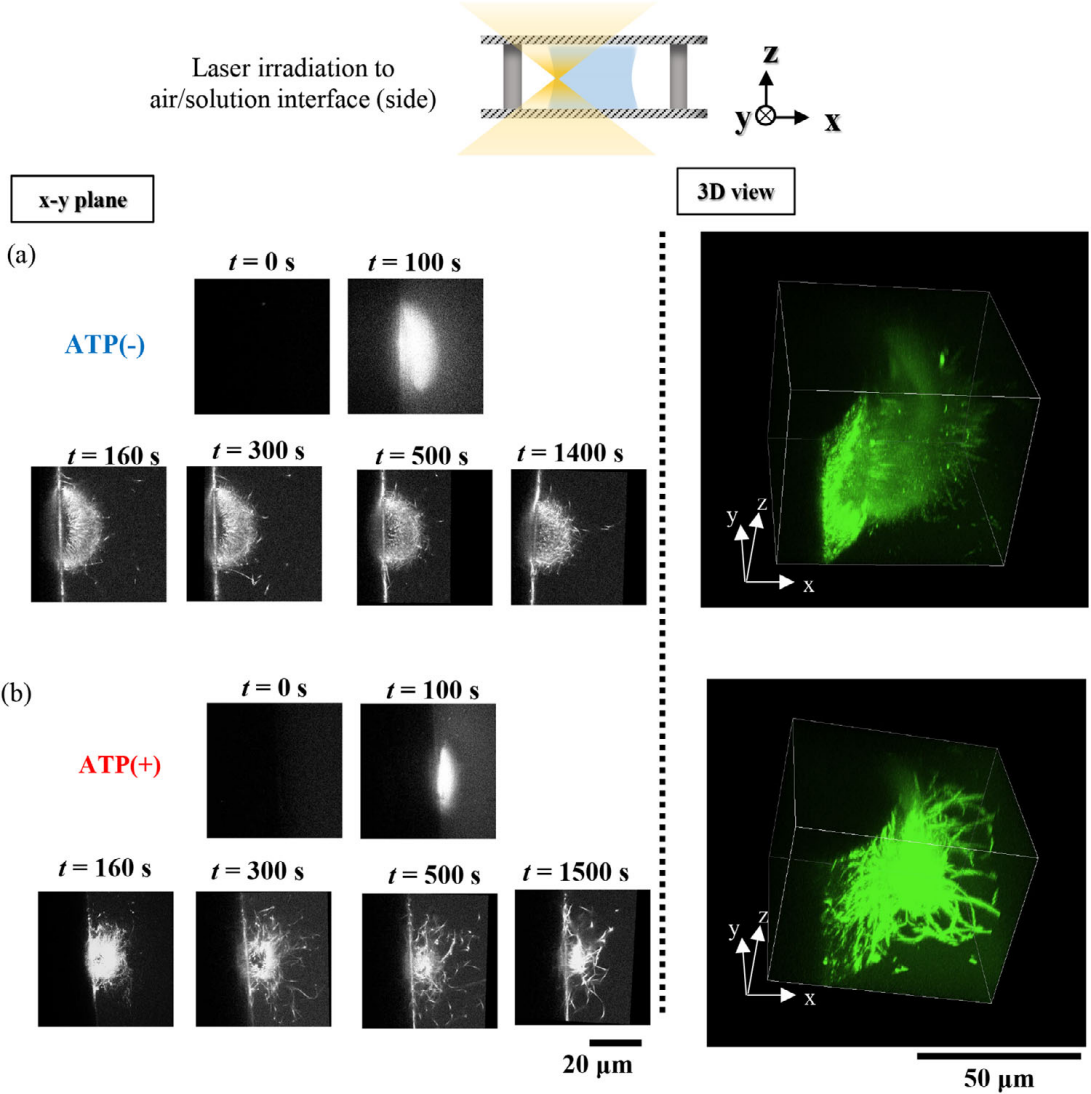
Fluorescence images of fiber aggregates formed by focused laser irradiation (∼0.8 W) at the air/solution interface (side). The fluorescence signal is originated from GFP‐fused kinesin. Laser irradiation began at t = 0 s and stopped at *t* = 100 s. The fiber aggregate was visualized in the absence (a) or presence (b) of ATP. Note that the 3D view in (a) was obtained in a separate experiment from the experiment used to obtain the data of the *x*‐*y* plane in (a).

To gain more insight into the dynamic motion of the laser‐generated fibers, we also obtained fluorescence images of the aggregates formed by focusing a laser on the side surface of a solution between two substrates, referred to as the air/solution interface (side). In the case of a solution containing tubulin and kinesin without ATP, a hemispherical aggregate formed at the interface (Figure 4a; Movie S8). After the laser irradiation was switched off, the increase in the size of the aggregate stopped, and its shape and size were almost unchanged even at *t* = 1400 s, which was also confirmed by imaging the 3D morphology of a laser‐generated aggregate with confocal microscopy. In contrast, the aggregate that was formed with kinesin and ATP showed dynamic movement of the fibers over 1200 s after the laser irradiation was switched off (Figure 4b; Movie S9). Confocal imaging revealed that long fiber bundles formed and were aligned toward the inner part of the solution.

**FIGURE 5:**
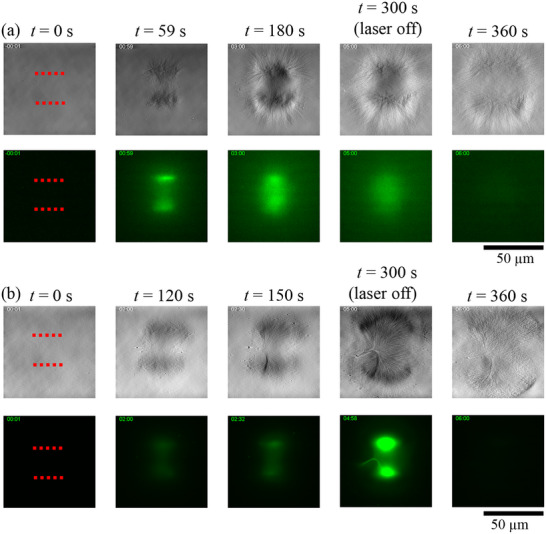
Fiber aggregates of tubulin and kinesin formed by scanning a focused laser beam in the (a) absence or (b) presence of ATP. Upper and lower images of (a) and (b) correspond to transmission and fluorescence images. The fluorescence signal is originated from GFP‐fused kinesin. Laser irradiation began at *t* = 0 s and stopped at *t* = 300 s at the air/solution interface (top). The red dotted lines correspond to the laser scanning.

The obtained results clearly show that highly ordered, dynamic microtubule assemblies can be formed by focused laser irradiation at air/solution interfaces. To verify the spatiotemporal controllability of the laser method, we demonstrated spatial patterning of dynamic microtubule assemblies by scanning a focused laser beam at an air/solution interface (top). Here, the focused laser beam was scanned as two parallel lines with a length of 25 µm and a gap of 25 µm, which partially mimics the geometry of the spindle apparatus of microtubules during cell division. In the case of tubulin and GFP‐fused kinesin without ATP, fibers formed within the scanning lines and grew toward the surroundings (Figure 5a; Movies S10 and S11). At approximately *t* = 1 min, fibers collided in the area between the two lines, but the orientation of each fiber was almost unchanged, thus maintaining the line‐symmetric shape of the fiber assemblies. In contrast, in the presence of ATP, the fiber orientation was significantly modulated by the collision (Figure 5b; Movies S12 and S13). From *t* ∼ 150 s, a single thick bundle formed and grew in one direction (toward the left side in Figure 5b), which caused the breakage of the symmetry of the assembly structures.

**FIGURE 6:**
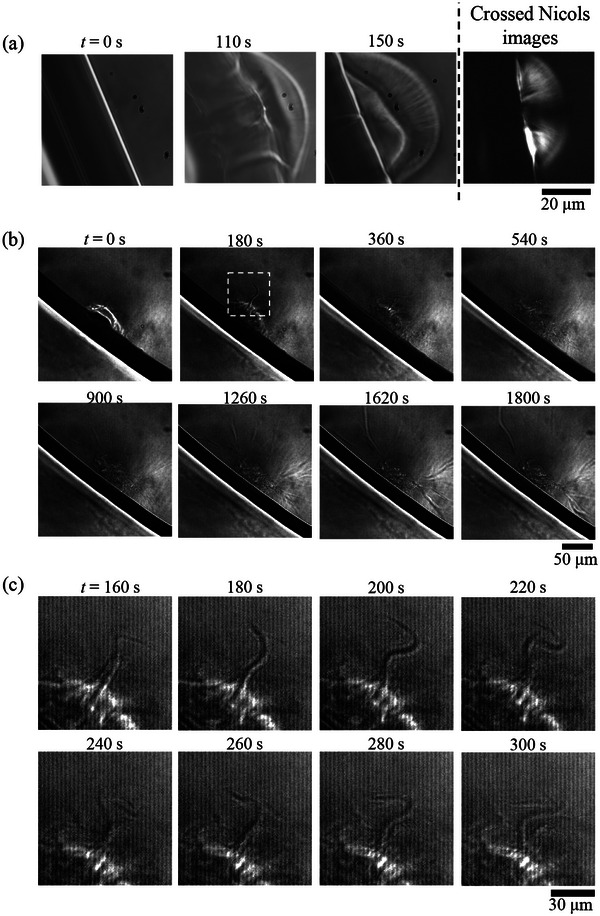
Images of fiber aggregates containing crosslinked tubulin. The hemi‐circular aggregate was formed by focused laser irradiation (1.0 W) at an air/solution interface (side) in the (a) absence or (b) presence of kinesin and ATP. (a) Laser irradiation began at *t* = 0 s and stopped at *t* = 150 s. The experiments for crossed Nicols imaging were separately performed from those for transmission imaging. (b) Laser irradiation with power of 1 W for 20 min was first performed, and then the images in (b) were taken after the laser beam was switched off. Magnified views of the white dotted box in (b) are shown in (c).

Finally, we also carried out laser experiments by using a solution containing crosslinked tubulin (Figure 6), which is known to form complex microtubule networks in which fibers are interconnected, which can modulate the motion of fiber assemblies [31]. First, in the case of a solution without kinesin and ATP, the laser irradiation at the air/solution interface resulted in the formation of a hemi‐circular aggregate (Figure 6a), which was also found for aggregates with inert tubulin (without crosslinking). The bright field and crossed Nicols images of the formed aggregate showed that the fibers were aligned in a radial or hemi‐circular manner. In the presence of kinesin and ATP, the hemi‐circular aggregates that were formed with chemically crosslinked tubulin also exhibited the dynamic motion and bundle formation after the laser irradiation was switched off (Figure 6b; Movie S14). In particular, by carefully observing the motion of each fiber, we found that fibers sometimes showed rotational or possibly helical motion similar to that of flagella (Figure 6c), which was not found for the solution with inert tubulin (Figures 4 and 5).

### 2.3 Mechanism of Dynamic Microtubule Assembly Formation by a Focused Laser Beam

We explored the underlying mechanism of dynamic microtubule formation using a focused laser beam. It is known that assembly of tubulins to microtubule is driven primarily by the hydrophobic effect, i.e., an entropy‐driven process [32, 33], in which binding sites of the tubulins have weak non‐covalent interactions. The formation of microtubules from tubulin is promoted by higher concentrations and temperatures (below the denaturation temperature) [17]. In this study, a focused laser beam at an air/solution interface locally increases the concentration and temperature at the laser focus through two photophysical effects: laser trapping by optical forces and heat generation by photoabsorption.

Laser trapping, often referred to as optical tweezers, uses a high‐NA objective lens to focus a laser beam into a solution, generating gradient forces that attract nanometre‐ and micrometre‐sized particles to the laser focus [18, 34]. The gradient force, typically in the fN to pN range, draws particles toward the laser focus, whereas the scattering force propels them along the beam propagation direction. Previous studies have demonstrated accumulation of biomolecules such as amino acids [35] and proteins (e.g., lysozyme, cytochrome *c*, and myoglobin) [23, 25, 26, 36] in solution using laser trapping, which enables the observation of crystallization and amyloid fibril formation. In contrast to these studies based on laser trapping within a solution, in our experiment, the laser beam was focused at an air/solution interface, leading to more stable protein trapping due to two factors. First, the amphiphilic nature of most water‐soluble proteins such as tubulin, promotes the formation of large clusters at an air‐solution interface. This tendency of proteins to form large clusters at an air/solution interface has been previously reported, particularly for those with amphiphilic properties, such as lysozyme [37]. Lysozyme molecules, driven by their amphiphilic nature, spontaneously adsorb on an air/solution interfaces and form various aggregated structures, including large clusters. These clusters, which are significantly larger than individual protein molecules, experience a stronger gradient force, leading to enhanced trapping and accumulation at the laser focus. Second, laser trapping at an air/solution interface restricts the *z*‐axis movement of trapped tubulins because of the scattering force, further enhancing the laser trapping stability. This stable protein trapping at an air/solution interfaces has been reported for α‐synuclein, in which the increased concentration led to amyloid fibril formation [27]. Thus, laser trapping at an air/solution interfaces provides a unique environment for concentrating tubulins, which plays a crucial role in the dynamic microtubule formation observed in this study.

In addition to the increase in concentration caused by laser trapping, the photothermal effects induced by the laser also contribute to microtubule formation. Water exhibits slight but nonnegligible photoabsorption of the 1064 nm laser due to the third harmonic vibration of the OH bond, leading to a local temperature increase at the laser focus. In this work, by using a temperature‐responsive fluorescence dye, we briefly estimated the averaged temperature rise in the area of the observation (∼200 µm × 200 µm) to be ∼20 K/W (Figure S3), which is comparable to that estimated by fluorescence correlation spectroscopy in the previous study (22–24 K/W at a laser focus) [24]. Thus the absolute temperature around a laser focus in the condition of this study (1 W laser irradiation) can be ∼313 K (20 K + room temperature of 293K). This suggests that the temperature around a laser focus does not reach the critical temperature of tubulin denaturation, ∼316–321 K or higher [38, 39], while proteins especially at the laser focus may partially be denaturated (e.g., insoluble component after the termination of laser irradiation shown in Figure 1d–f) and act as nucleation centers. In addition to the molecular accumulation by laser trapping, the localized heating with a focused laser beam should further enhance microtubule formation by increasing the kinetics of tubulin polymerization. The impact of this laser‐induced heating was experimentally confirmed by varying the ratio of D_2_O to H_2_O in the solution. D_2_O has a smaller extinction coefficient at 1064 nm (*α* = 0.012 cm^−1^) than H_2_O (*α* = 0.14 cm^−1^), resulting in less absorption and reduced heating. As expected, an increase in the D_2_O ratio (Figure S4) led to smaller aggregate sizes and longer formation times, indicating that the temperature increase contributed to the aggregation process. It should be also noted that the fiber alignment under the higher D_2_O ratio condition was not prominent compared to that with pure H_2_O solvent (Figure 1). This suggests that thermal convection may be involved in the formation of highly ordered fiber networks, a similar phenomenon was also observed in other studies regarding the alignment of microtubules in liquid flow chambers without the use of lasers [40, 41]. However, the observed microtubule formation in unsaturated tubulin solutions (0.2 µm, Figure 1d–f) cannot be attributed solely to the laser‐induced temperature increase. Even with a 25 K temperature increase, the 0.2 µm tubulin concentration remains below the critical concentration for microtubule formation at 45°C (25 K higher than the initial solution temperature of 20°C) [17]. This observation further supports the crucial role of laser trapping in accumulating tubulin molecules at an air/solution interface. The trapping force effectively maintains the formation of microtubule assembly at the laser focus, as evidenced by the firm localization of assemblies during laser irradiation and subsequent diffusion upon laser termination (Figure 1). Moreover, the laser trapping can lead to the accumulation of not only individual tubulin dimers but also larger molecular clusters formed by laser‐induced temperature increase. This synergistic interplay between laser trapping and photothermal effects is further supported by the findings of previous studies, in which the combination of UV and IR laser irradiation promoted photopolymerization by efficiently trapping oligomers created by UV irradiation [42]. Therefore, we conclude that the two distinct photophysical effects, namely, the increase in local concentration caused by laser trapping and the increase in temperature by photoabsorption, act in concert to promote the formation of microtubule assemblies from tubulin solutions at an interface between air and H_2_O‐based solutions, which is under the conditions more similar to physiological conditions (without the use of specific photochemical reactions and deuterated water). This cooperative mechanism underlies the efficient and dynamic microtubule formation observed in our study.

The dynamic motion of the laser‐formed microtubule assemblies in the presence of motor proteins and ATP clearly revealed that the biological activities of proteins (i.e., tubulin and kinesin) are retained upon focused laser irradiation, while the TEM images showed amorphous‐like objects due to denaturation may also form in addition to microtubule fibers (Figure 2). Interestingly, the laser‐formed microtubules showed radial alignment from the laser focus, which is likely the origin of the translational or rotational motion of the microtubule assemblies in the presence of kinesin and ATP. In addition, the formation of thick microtubule bundles during the directed motion (Figure 3b) can be attributed to the driving interactions of kinesins with aligned microtubules, as localization of GFP‐fused kinesins on microtubules was clearly observed in Figure 4b. This behavior is in contrast to that the case of the solution without ATP (Figure 4a), in which static semi‐spherical assemblies with thinner fibers formed. This fine‐tuned spatiotemporal controllability of the formation of dynamic microtubule assemblies with a focused laser beam enabled us to investigate the structure‐motion relationships through various biologically mimicked patterns (Figure 5). Furthermore, as our laser method is based on photophysical effects, it can be combined with the molecular engineering approaches, which as demonstrated by the emergence of flagellar‐like movement with crosslinking of microtubules in this study (Figure 6c). Here, the TEM images of aggregates containing crosslinked tubulin (Figure S5) showed the characteristic ring shapes of microtubules in the cross‐sectional image, which are similar to those observed for non‐crosslinked microtubules in Figure 2. This indicates that crosslinking itself does not disturb the formation of microtubule structures, probably due to a low ratio of crosslinked tubulin (4 mol%) with respect to total tubulin. On the other hand, in the presence of motor proteins and ATP, we previously found that a microtubule network with sparse crosslinking on a kinesin‐functionalized glass substrate does not always undergo the long‐distance and straight longitudinal movements, but frequently change their directions and makes vibrational or zigzag motions of the network as a whole [31]. In contrast, non‐crosslinked microtubule fibers prefer to undergo linear longitudinal displacement and the lateral movement is strongly restricted. Since flagellum‐like rotation should not be expressed solely by linear longitudinal displacement but by the coupling of longitudinal and lateral movements (e.g., zigzag motions), sparse crosslinking that can mechanically connect microtubules should play a crucial role in exhibiting complex dynamic motions such as flagellum‐like rotation. In fact, Sanchez et al. also reported microtubule bundles where kinesin clusters bind and walk along two neighboring microtubules could exhibit beating patterns reminiscent of those found in eukaryotic cilia and flagella [15]. We expect further fine manipulation of laser‐induced aggregation will lead to more controllable assembly of various motile structures with crosslinking effects in the future.

## 3 Conclusion

We demonstrated spatiotemporal control of the formation of highly ordered and dynamic microtubule assemblies with a focused laser beam. The local increases in the concentration and temperature induced by photophysical effects can spatiotemporally control the accumulation of proteins without the use of photochemically active crosslinkers, which provides another clue to fabricating complex networks of biomolecules that can mimic various cellular structures. It is worth noting that our photophysical approach does not require any chemical modification of the molecules, enabling one to use native biomolecules (such as tubulin, kinesin, and ATP). This point is particularly important to avoid undesired loss of biological functions of native biomolecules by chemical modifications. Furthermore, since our photophysical method relies on the use of near‐infrared lasers, it does not typically interfere with the excitation wavelengths of light sources used for fluorescence imaging (e.g., in the ultraviolet to visible light range), whereas conventional photochemical methods tend to do. Nevertheless, aside from the respective advantages of each approach, it should be also emphasized that our photophysical methods can also be combined with conventional photochemical techniques because the working mechanisms are fundamentally different. Thus, one can expect that our photophysical method can serve as another, independent control means, which enables one to reconstruct complex structures of protein assemblies by combinational use with photochemical methods. We foresee that this laser method with greater flexibility can produce various biologically mimicked patterns, which will extend biomaterial engineering toward the fabrication of more complex structures and provide fundamental insights into the structure‐function relationships of biomolecular assemblies.

## 4 Experimental Section

### 4.1 Purification of Tubulin and Kinesin

Tubulin was extracted from porcine brains in a high‐concentration piperazine‐N, N'‐bis (2‐ethanesulfonic acid) (PIPES) buffer [1 m PIPES, 20 mm ethylene glycol bis(β‐aminoethyl ether)‐N, N, N', N'‐tetra acetic acid (EGTA), 10 mm MgCl_2_, pH adjusted to 6.8 using KOH [43]. The purified tubulins were handled in buffers based on a standard buffer called ‘BRB’ [80 mm PIPES, 1 mm EGTA, 1 mm MgCl_2_, pH 6.8] with some modifications as described below. GFP‐fused kinesin (K560‐GFP), consisting of the first 560 amino acids of kinesin‐1, was obtained by modifying some steps of the original purification method established by Vale's group [31, 44]. In brief, *E. Coli* strain BL21(DE3) transformed with the plasmid DNA was cultured in 0.5 L medium to OD600 of 1 and protein expression was induced by the addition of 0.1 mm IPTG for more than 9 h. The harvested cells were lysed by sonication, and the proteins were initially purified by Ni‐His affinity. Active fraction of K560‐GFP was further purified through microtubule‐affinity spin down in which microtubule‐binding proteins in the absence of ATP and released proteins with ATP were collected. The aliquots were quick frozen by liquid nitrogen and stored at −80°C until use. The plasmid construct of K560‐GFP was generously provided by R. D. Vale and M. Tomishige. This experimental process was approved by the Committee for Safe Handling of Living Modified Organisms in Saitama University (Permission numbers:H26‐1, R6‐G‐1) and carried out according to the guidelines of the committee. The tubulin concentration was determined by measuring the absorbance at 280 nm and calculated from the absorption coefficient. The K560‐GFP concentration was determined by densitometry using sodium dodecyl sulfate polyacrylamide gel electrophoresis (SDS‐PAGE) images of Coomassie Brilliant Blue‐stained, samples and a calibration curve with bovine serum albumin as the reference protein.

### 4.2 Preparation of Chemically Modified Tubulins

Fluorescently labelled tubulins were prepared by modifying tubulins with Alexa Fluor 488 NHS ester (A20000, Invitrogen) according to the established method [45, 46]. In brief, tubulin was polymerized at first, and the labelling reaction followed. After the reaction, Alexa 488‐labelled tubulins which can polymerized and depolymerize in appropriate conditions were purified by repeating warm‐ and cold‐spin; warm‐spin is to collect the sedimented microtubules and cold‐spin is to collect depolymerized tubulins in the supernatant after ultracentrifuges. The aliquots were quick frozen by liquid nitrogen and stored at −80 C until use. The labeling ratio was determined based on the UV/visible light absorbances of tubulin protein and Alexa 488 and their extinction coefficients of 115 000 m ^−1^cm^−1^ at 280 nm and 71 000 m ^−1^cm^−1^ at 495 nm, respectively. The labelling ratio was measured to be Alexa 488 / tubulin = 0.48 at a molar ratio for 150 µm tubulin solution, and the labelling ratio was adjusted to 0.025 for 100 µm tubulin through mixing with purified unlabeled tubulins.

The cross‐linking reaction of tubulins was typically performed in a 100 µL‐volume containing 100 µm tubulin, 80 mm PIPES (pH 6.8 by KOH), 1 mm EGTA, 6 mm MgCl_2_, 5 mM guanosine‐5'‐triphosphate (GTP), and 50 µM *bis*‐*N*‐hydroxysuccinimidyl ester polyethylene glycol (bis‐NHS PEG, MW 3917, SUNBRIGHT DE‐050GS, NOF); the molar ratio was set to NHS‐ester group / tubulin = 1. To avoid inactivation of tubulins by modification of the critical site for polymerization, i.e., the tubulin‐tubulin interface, the tubulins were initially polymerized into microtubules at 37°C for 30 min before the cross‐linker solution was added as well as the case of fluorescent labelling [45, 46]. Then, 5 µL of 1 mm bis‐NHS‐PEG was added to finalize the above‐described conditions for cross‐linking and mixed well; bis‐NHS‐PEG was solubilized in water immediately before its addition owing to its short hydrolysis life. After 1 h of cross‐linking reaction keeping at 37°C, the cross‐linked microtubules were depolymerized into tubulins by cooling the solution on ice. The samples containing dimerized tubulins were aliquoted and flick‐frozen by liquid nitrogen for storage at −80°C until use. With this method, 4% of the tubulins should be dimerized by crosslinking according to a previous report, in which the dimerization ratio was probed by SDS‐PAGE and densitometry [31].

### 4.3 Optical Setup

Figure S6 shows a schematic illustration of the optical setup for single‐point irradiation (Figures 1, 2, 3, 4, and 6). As a laser source for microtubule formation, a continuous wave Nd^3+^:YAG laser (λ = 1064 nm, BL‐106SU‐FE, Spectra Physics or MATRIX 1064‐10‐CW, Coherent) was used and introduced into a laser‐scanning confocal microscope (A1R MP+, A1, or C2 Nikon) or an inverted microscope (IX‐71, OLYMPUS). The laser was focused onto a sample through an objective lens (60×, NA = 0.90, UPLFLN, OLYMPUS or 40×, NA = 0.95, PLANAPO). The laser power was adjusted by a half‐wavelength plate (coupled with a polarizing beam splitter) with being measured with a power meter (Vega, Ophir Optronics) on the stage. A blue laser (λ = 488 nm) was used as the excitation light source for GFP fused to kinesin, and fluorescence images were acquired with high‐sensitivity detectors equipped in a confocal microscopy system.

Pattern‐scanning of focused laser beam was performed using a Tweez305 laser tweezer system (Aresis) with a 5 W‐continuous‐wave laser and was observed with a combined laser‐scanning confocal system (A1R MP+, Nikon) based on an inverted microscope (Ti2‐E, Nikon) using a water immersion objective lens (Plan apo, 60×, NA1.2, Nikon) (Figure S7). For the objective, D_2_O was used as immersion water to avoid the energy absorption of 1064 nm‐laser light by H_2_O. The laser power for optical trapping was tuned to 300 mW through the objective lens. The scanning for optical trapping along the pattern was enabled by the acousto‐optical deflector (AOD) included in the Tweez305 system. To scan a pattern of a pair of parallel lines (Figure 5), the switching rate of the scanning was set to 100 kHz, which was the maximum of the device so that the time lag to switch from one line to another was minimized. For the pair of lines that were 25 µm in length and 25 µm apart, the focused laser beam was scanned for 210 s.

### 4.4 Estimation of Temperature Increase by Laser Irradiation

To estimate temperature, increase by laser irradiation, a polymeric fluorescence dye (Poly(NNPAM‐co‐APTMA‐co‐DBThD‐AA‐co‐BODIPY‐AA, Cellular Thermoprobe for Fluorescence Ratio, Funakoshi) was used. This dye can work as a sensitive fluorescent thermometers that employ a fluorescence ratio at two different wavelengths, for instance, the fluorescence intensity ratio at 580 nm (corresponding to the environment‐sensitive DBThD‐AA units) to 515 nm (corresponding to the environment‐insensitive BODIPY‐AA units) is strongly correlated with temperature. We first prepared a 4‐(2‐hydroxyethyl)‐1‐piperazineethanesulfonic acid (HEPES) buffer solution (pH 7.8) containing 1 mg/mL of the fluorescence dye. Then an aliquot (10 µL) of the solution was added to a plastic container with a glass cover slip (glass bottom dish, Iwaki). The container was placed in a holder of a microscope stage with a temperature control unit (Tokai Hit). Then fluorescence signals of green (λ = 500–550 nm) and red (λ = 573–617 nm) colours were simultaneously measured by using the above‐mentioned laser‐scanning confocal microscope (C2 Nikon). Temperature under laser irradiation was determined from the obtained green‐red fluorescence intensity ratio (*I*_573 − 617 *nm*_ / *I*_500 − 550 *nm*_) according to calibration curves that were separately made by changing the temperature without laser irradiation. In this work, to avoid the undesired photo‐bleaching or fluorescence via two‐photon excitation with s focused laser beam of relatively high power (1 W) that disturbs the precise temperature measurement, we first determined the temperature increase by the laser irradiation with the power of 0.1 W, which is lower than that used for tubulin experiments (1 W). Since the temperature increase by laser irradiation is generally proportional to laser power [47], we can estimate the temperature increase at 1 W by multiplying the measured temperature by ten.

### 4.5 Formation of Fiber Aggregates by Focused Laser Irradiation

In the initial study (Figure 1), the formation of fiber aggregates by focused laser irradiation was probed under a confocal laser scanning microscope by using thawed 100 µm Alexa 488‐labelled tubulin (AT) with appropriate dilutions on ice. At the final concentration, 0.2 or 20 µm AT (Alexa 488/tubulin = 0.025) was prepared in BRB buffer supplemented with∼ 3 mm MgCl2, ∼1 mm GTP, 1 mm tris(2‐carboxyethyl)phosphine (TCEP), 120 U mL–1 catalase, 6 U mL–1 glucose oxidase, and 15 mm D‐glucose; an oxygen scavenging enzymatic system was introduced to prevent fading of the fluorescent probes. Water was used as a solvent for the buffer except for in the experiments with deuterated water (99.9%, Sigma‒Aldrich) (Figure S4).

The sample solution with a volume of 10 µL was dropped onto a glass coverslip (24 × 40 mm, thickness: 0.13–0.17 mm, C024401, Matsunami) hydrophilized with a surfactant (Hellmanex III, Hellma Analytics) and sealed within a chamber made of a silicone rubber sheet (thickness: 2 mm, 70336‐20, Electron Microscopy Sciences) as a spacer and of another glass coverslip for the top. Hydrophilization of the glass substrate was performed in advance by immersing the coverslips in 1% Hellmanex III for one day. After 3 cycles of washing via 10‐min of sonication in ultrapure water with water exchange, the coverslips were well dried by blowing nitrogen gas before use. The sample chamber containing AT solution was placed on the microscope stage, and formation of fiber aggregations was induced by irradiation with a focused laser beam under the optical setup described in the “Optical setup” section above.

### 4.6 TEM Imaging of Aggregated Fibers

TEM images (Figure 2) were obtained to confirm the formation of microtubules by visualizing their tubular structure in the aggregates formed by the laser. The samples for TEM observation were prepared as described below. A glass surface was coated with kinesin to immobilize the formed fibrous aggregates on the glass substrate so that they would not diffuse into the solution. Kinesin coating was performed by the following method. In this study, GFP‐fused kinesin (K560‐GFP) was used, and anti‐GFP (0.2 mg mL–1 in BRB), which binds specifically to GFP, was dropped onto the glass surface and allowed to stand for 15 min. To prevent binding of kinesin to the uncoated area of the anti‐GFP antibody, casein (0.5 mg mL–1) was added, and the mixture was allowed to stand for 10 min. Finally, the glass surface was washed with BRB. Ten microlitres of 20 µm native tubulin solution in BRB buffer with 2 mm MgCl2 and 1 mM GTP was added, and a laser beam was focused on the glass/liquid/air three‐phase interface to induce the formation of tubulin aggregates.

Furthermore, the aggregated protein samples were fixed to prevent degradation due to autolysis and putrefaction. To fix the proteins, 0.8 µL of 25% glutaraldehyde and 1.7 µL of 25% tannic acid were added to the 10 µL‐sample droplet, which was left for 1 h. After the supernatant was removed, a drop of 1% osmium tetroxide solution was added, and the mixture was allowed to post‐fix for 30 min. The samples were then embedded in epoxy resin. Since epoxy resin is hydrophobic, the samples were dehydrated by exposing them to 50%‐60%‐70%‐80%‐90%–95% EtOH for 5 min each. Finally, the samples were exposed to 100% EtOH for 5 min, and this process was repeated three times. The EtOH was then replaced with Quetol‐651 (Nisshin‐EM); the samples were exposed to 25%‐50%‐75%–100% Quetol‐651 for 15 min each, and the supernatant was removed. Finally, the samples were left in an oven at 60°C for two days to allow the polymerization of the resin to completely proceed. The sliced sections with a thickness of ∼80 nm were observed by TEM (Hitachi, H‐7500) [48, 49].

### 4.7 Formation of Fiber Aggregate in the Presence of Kinesin

For the experiment shown in Figures 3 and 4, a solution mixture of native tubulin and K560‐GFP was prepared on ice to yield a final concentration of 20 µm tubulin and 8.4 nm K560‐GFP in BRB buffer supplemented with 4 mm MgCl2, 1 mm GTP, 1 mm ATP, 0.7 mm TCEP, 40 U mL^−1^ catalase, 2 U mL^−1^, glucose oxidase, and 5 mm D‐glucose. A sample droplet of 10–15 µL was placed on a hydrophilized glass coverslip to allow observation of the gas/liquid interface at the top of the thin droplet; the inner geometry of the chamber, which depended on the shape of the silicone rubber spacer was 20 mm in diameter and 0.5 mm thick, which a droplet height of 120–160 µm and a spread shape sufficiently smaller than the diameter. For observation at the side of the droplet, the sample solution was dropped onto an untreated glass substrate, and the solution was sandwiched between cover glasses with a 100‐µm‐thick silicon rubber sheet as a spacer to ensure that the droplet contacted both the top and bottom coverslips. The samples were sealed in the chambers at room temperature and placed on the microscope stage for laser irradiation.

### 4.8 Spatial Patterning of Fiber Aggregates With Kinesins by Laser Scanning

For the experiment shown in Figure 5, a solution mixture of 20 µm unlabeled tubulin and 150 nm K560‐GFP in BRB buffer was prepared with supplementation of 3 mm MgCl_2_, 1 mm GTP, 3 mm ATP, 1 mm TCEP, 120 U mL^−1^ catalase, 6 U mL^−1^ glucose oxidase, and 15 mm D‐glucose. For the sample chamber including a droplet of the sample solution (10 µL) on a hydrophilized coverslip, focused laser irradiation at the top of a droplet was performed following the scanning patterns described in the “Optical setup” section below.

### 4.9 Laser‐Induced Fiber Aggregation With Crosslinker

The chemically crosslinked tubulin (CT) that contained 100 µm tubulin was diluted with BRB buffer 5‐fold to obtain a final concentration of 20 µm tubulin including 4% crosslinked dimers in BRB supplemented with 2 mm MgCl_2_ and 1 mm GTP. For the case of fiber aggregation in the presence of kinesin and ATP, a solution mixture was prepared as follows: 20 µm CT, 2.6 mm MgCl_2_, 1 mm GTP, 1 mm ATP, 0.7 mm TCEP, 40 U mL^−1^ catalase, 2 U mL^−1^ glucose oxidase, and 5 mm D‐glucose. The sample droplet was placed on a hydrophilized coverslip and sealed in a chamber for focused laser irradiation and observation (Figure 6).

### 4.10 Image Analysis and Adjustment

ImageJ (NIH) was used for length/area measurement, background management, brightness and contrast adjustment of images and movies shown in this work. Artificial fringe noise in images and movies that were obtained with a confocal microscope (e.g., Movie S15 that corresponds to Figure 1a) were removed via fast Fourier transform (FFT) functions of ImageJ.

## Conflicts of Interest

The authors declare no conflicts of interest.

## Supporting information




**Supporting File 1**: advs75531‐sup‐0001‐SuppMat.pdf.

**Supporting File 2**: advs75531‐sup‐0002‐Movie S1.mp4.

**Supporting File 3**: advs75531‐sup‐0003‐Movie S2.mp4.

**Supporting File 4**: advs75531‐sup‐0004‐Movie S3.mp4.

**Supporting File 5**: advs75531‐sup‐0005‐Movie S4.mp4.

**Supporting File 6**: advs75531‐sup‐0006‐Movie S5.mp4.

**Supporting File 7**: advs75531‐sup‐0007‐Movie S6.mp4.

**Supporting File 8**: advs75531‐sup‐0008‐Movie S7.mp4.

**Supporting File 9**: advs75531‐sup‐0009‐Movie S8.mp4.

**Supporting File 10**: advs75531‐sup‐0010‐Movie S9.mp4.

**Supporting File 11**: advs75531‐sup‐0011‐Movie S10.mp4.

**Supporting File 12**: advs75531‐sup‐0012‐Movie S11.mp4.

**Supporting File 13**: advs75531‐sup‐0013‐Movie S12.mp4.

**Supporting File 14**: advs75531‐sup‐0014‐Movie S13.mp4.

**Supporting File 15**: advs75531‐sup‐0015‐Movie S14.mp4.

**Supporting File 16**: advs75531‐sup‐0016‐Movie S15.mp4.

## Data Availability

The data that support the findings of this study are available from the corresponding author upon reasonable request.
