# Supplementary material for: Spatiotemporal Control of Formation of Dynamic Protein Fiber Assemblies via Photophysical Effects of a Focused Laser Beam
Source: Adv Sci (Weinh). 2026 May 17;13(40):e75531. doi: 10.1002/advs.75531 (PMC13335755; doi:10.1002/advs.75531)
Supplement: Supplementary file 1 — Supporting File 1: advs75531‐sup‐0001‐SuppMat.pdf. [file ADVS-13-e75531-s004.pdf]

Supporting Information

Spatiotemporal Control of Formation of Dynamic Protein Fiber Assemblies via Photophysical Effects of a Focused Laser Beam

Hiroshi Y. Yoshikawa,[a]*, Ren Shirata,[a,b] Takuya Takeshige,[b] Riki Yoshida[a], Fumika Kiryu,[b,c] Kei Takano,[b] Shuma Matsumoto,[a] Reiji Kawanami,[a] Natsumi Sawada,[b] Chi-Shiun Wu,[b,c,d] Yang-Hsin Shih[c], Hiromasa Niinomi[e], Takahisa Matsuzaki[a], Seiichiro Nakabayashi[b], Tomoaki Matsuura,[f] Teruki Sugiyama[c,g], Ryuzo Kawamura[b]*

[a] Graduate School of Engineering, The University of Osaka, 2-1 Yamadaoka, Suita, Osaka 565-0871, Japan

[b] Department of Chemistry, Saitama University, Shimo-okubo 255, Sakura-ku, Saitama 338-8570, Japan

[c] Department of Applied Chemistry, National Yang-Ming Chiao Tung University, Hsinchu, 30010, Taiwan

[d] Department of Applied Chemistry, National Chiayi University, Chiayi 60004, Taiwan

[e] International Institute for Sustainability with Knotted Chiral Meta Matter, Hiroshima University, Hiroshima 739-8531

[f] Earth-Life Science Institute, Institute of Science Tokyo, Ookayama 2-12-1, Meguro-Ku, Tokyo 152-8550, Japan

[g] Division of Materials Science, Graduate School of Science and Technology, Nara Institute of Science and Technology, Ikoma 630-0192, Japan

*Double corresponding authors;

Email: hiroshi@ap.eng.osaka-u.ac.jp Tel: +81-(0)-6-6879-4909

Email: ryuzo@mail.saitama-u.ac.jp Tel: +81-(0)-48-858-3383

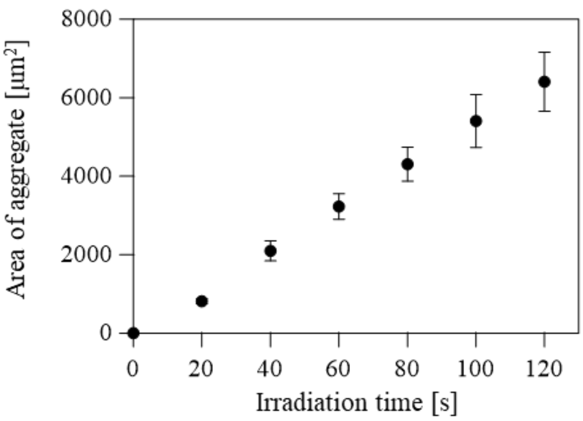

Figure S1. Average area of the laser-formed aggregates as a function of time. The experimental conditions were the same as those in Fig. 1a.

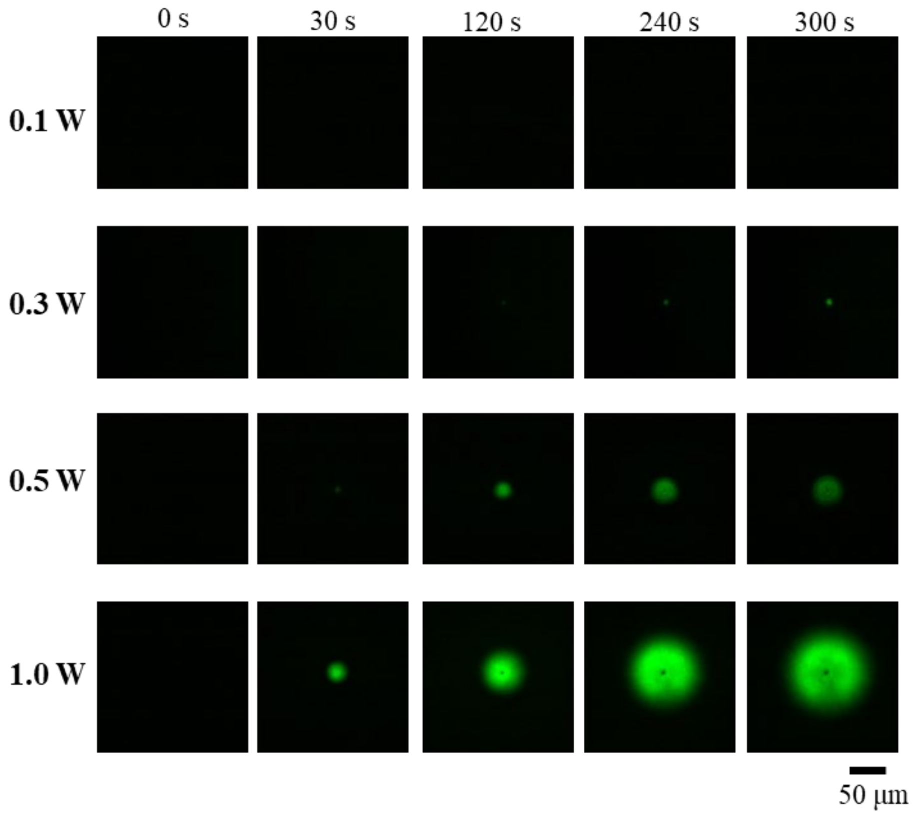

Figure S2. Laser power dependence of formation of fiber aggregates (initial tubulin concentration: 20 μM) by focused laser irradiation at an air/solution interface (top). Laser irradiation began at $t = 0$ s and stopped at $t = 300$ s.

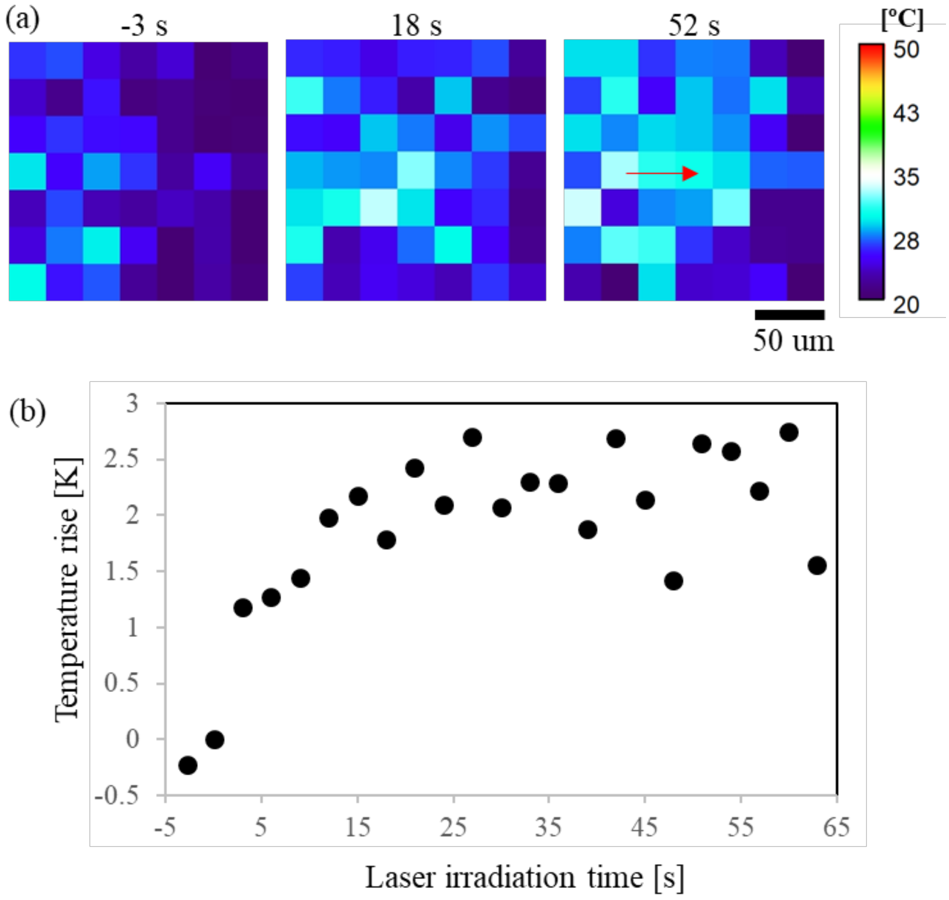

Figure S3. Estimation of temperature increase induced with a focused laser beam. (a) Spatial distribution of temperature at and around a laser focus with the laser power of 0.1 W. The laser irradiation was initiated from 0 s. The red arrow represents a laser focus. (b) the average temperature rise in the observation area (~ 200 μm × 200 μm) as function of laser irradiation time. According to the linear relationship between temperature rise and laser power,[1] one can estimate the temperature rise with the laser power of 1 W to be approximately ~ 20 K (~ 2 K × 10).

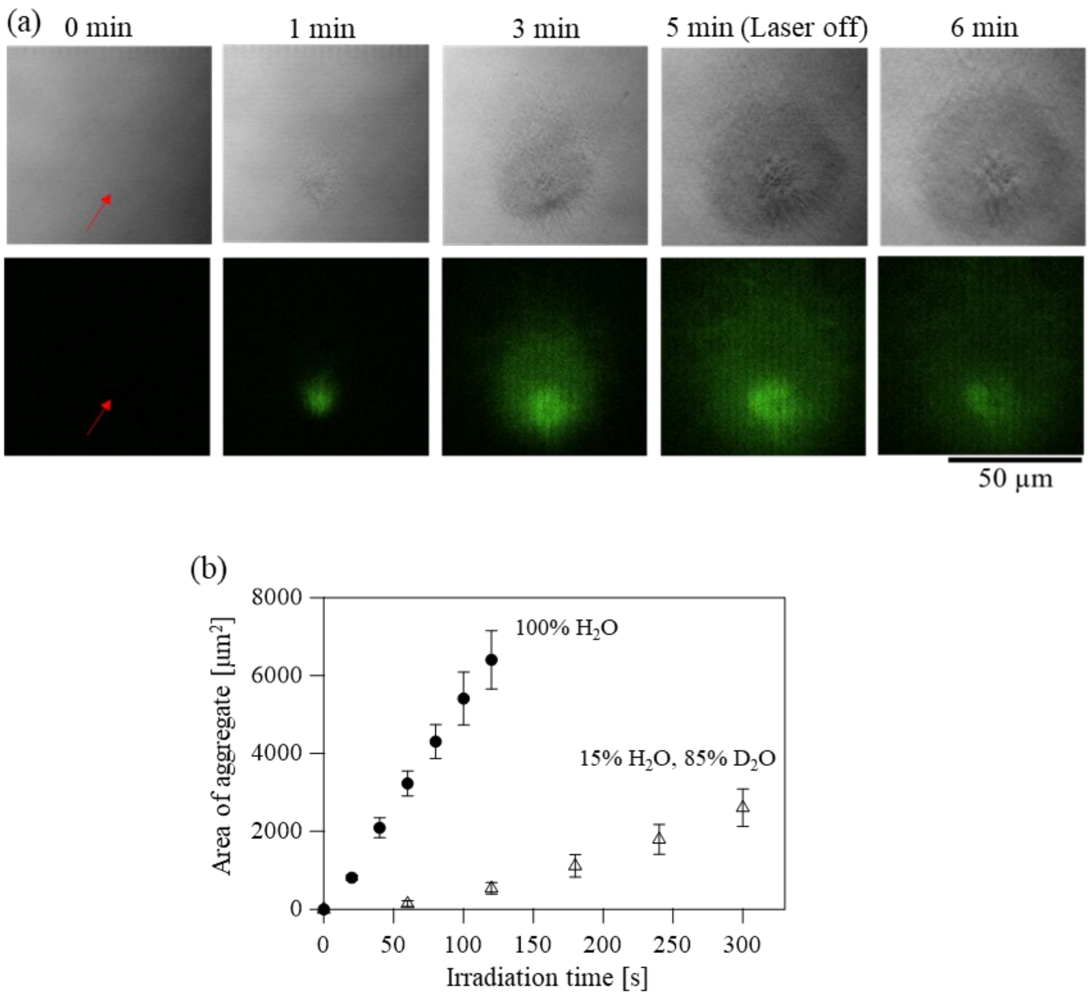

Figure S4. (a) Sequential images of the formation of a microtubule aggregate in the solution with a mixture of water (15% v/v) and deuterated water (85% v/v) as a solvent by focused laser irradiation (1.0 W). The laser focus was set at the air/solution interface (top). The arrows represent the laser focus positions. (b) Average area of the laser-formed aggregates as a function of time. Note that the data points for 100% H_2O are the same as those in Fig. S1.

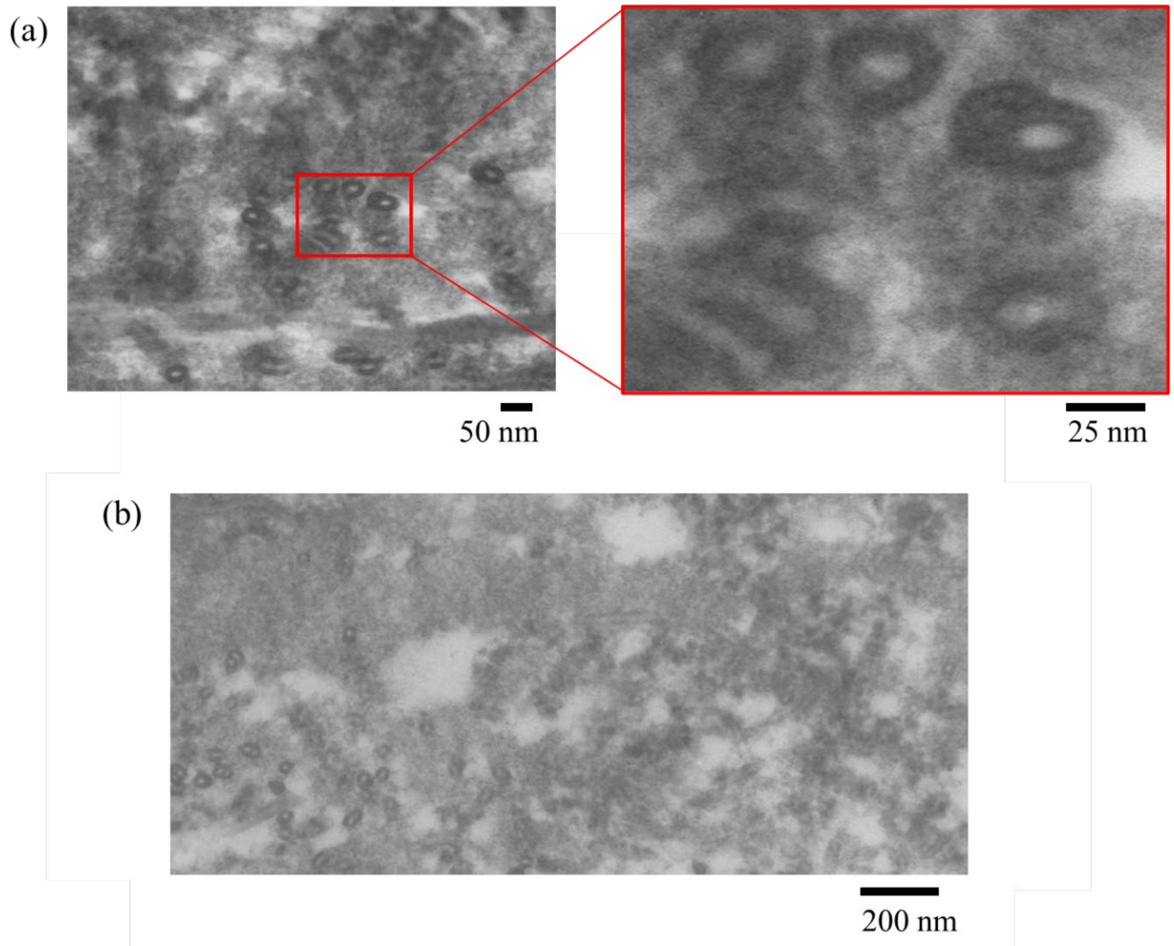

Figure S5. TEM images of the fiber aggregate containing crosslinked tubulins, which were made by chemical crosslinking with bis-NHS PEG during microtubule formation at 37˚C and disassembled to tubulin form by chilling on ice.

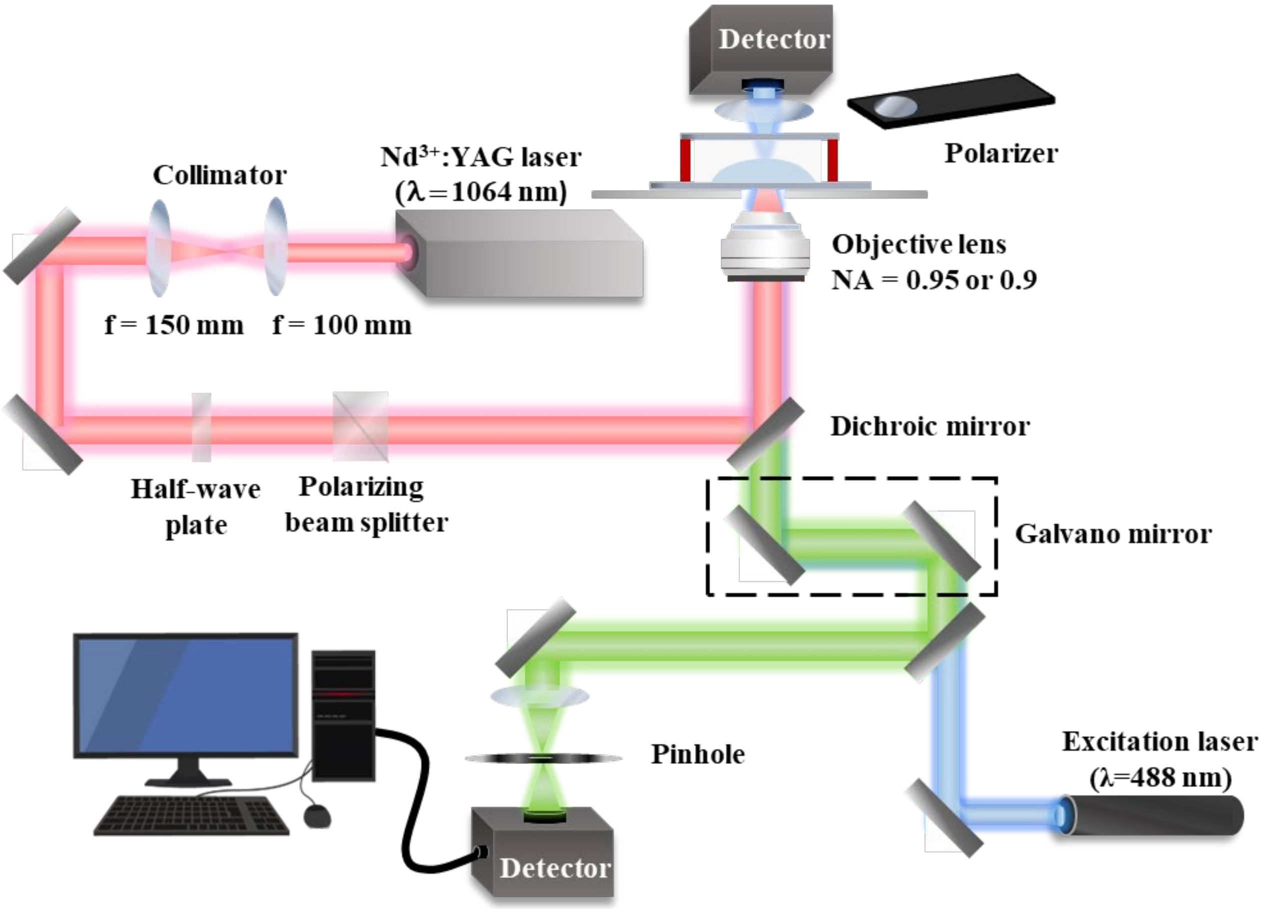

Figure S6. Optical setup of the single-point irradiation experiments. The data in Figs. 1, 2, 3, 4, and 6 were obtained with this setup.

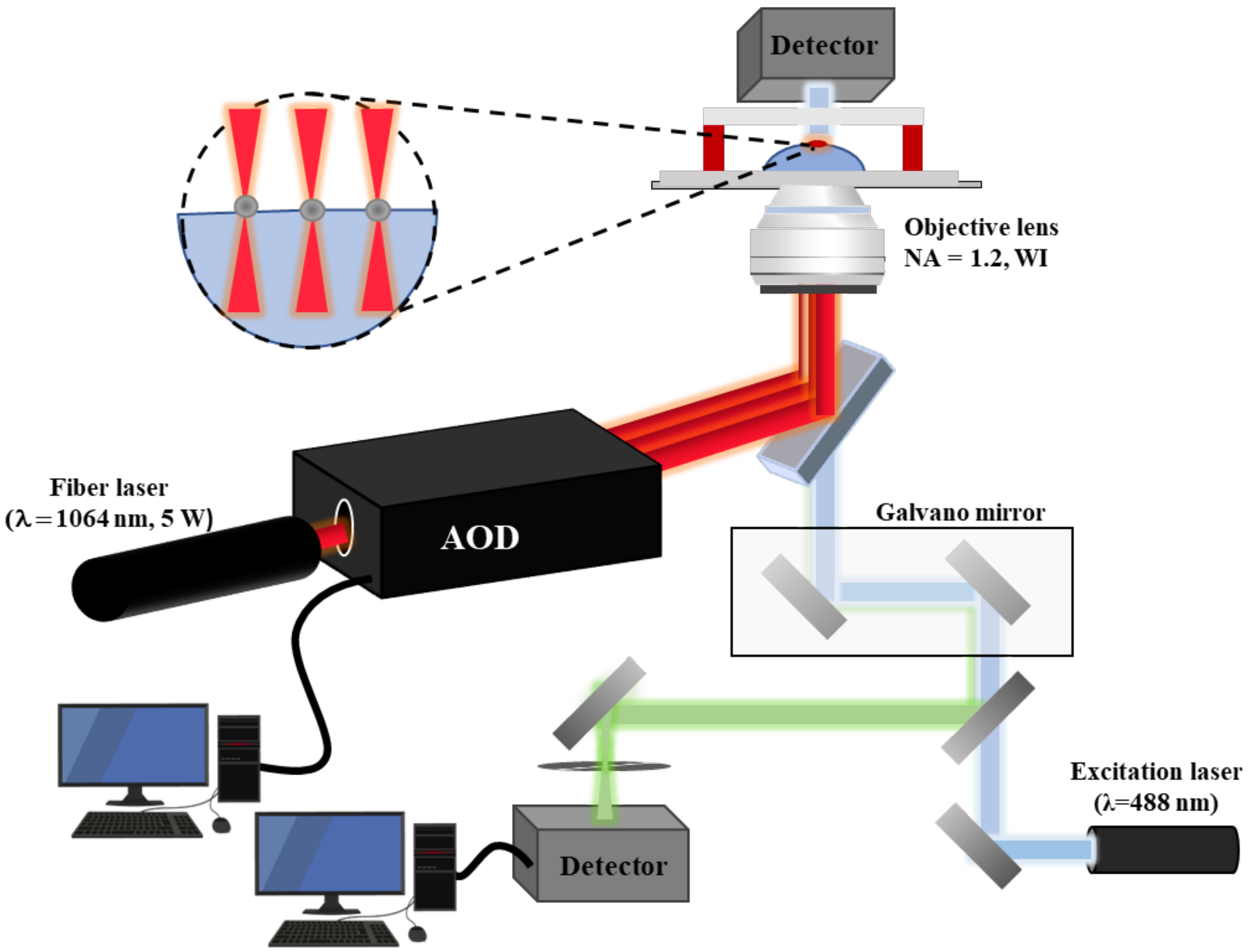

Figure S7. Optical setup of the scanning irradiation. The data in Fig. 5 were obtained with this setup.

Description of Supporting Movies

File Name: Supplementary_Movie1
Description: The movie of transmission imaging corresponds to Fig.1a.

File Name: Supplementary_Movie2
Description: The movie of fluorescent imaging corresponds to Fig.1b.

File Name: Supplementary_Movie3
Description: The movie of crossed Nicols imaging corresponds to Fig.1c.

File Name: Supplementary_Movie4
Description: The movie of transmission imaging corresponds to Fig.1d.

File Name: Supplementary_Movie5
Description: The movie of fluorescent imaging corresponds to Fig.1e.

File Name: Supplementary_Movie6
Description: The movie of crossed Nicols imaging corresponds to Fig.1f.

File Name: Supplementary_Movie7
Description: The movie of fluorescence imaging corresponds to Fig. 3b. Playback speed was increased by factor of one hundred eighty, namely, the movie is 30 mins long when converted to real time. The time stamps of key snapshots are shown in Fig. 3b.

File Name: Supplementary_Movie8
Description: The movie of fluorescence imaging corresponds to lower images of Fig. 4a. Playback speed was increased by factor of one hundred eighty, namely, the movie is 30 mins long when converted to real time. The time stamps of key snapshots are shown in Fig. 4a.

File Name: Supplementary_Movie9
Description: The movie of fluorescence imaging corresponds to lower images of Fig. 4b. Playback speed was increased by factor of one hundred eighty, namely, the movie is 30 mins long when converted to real time. The time stamps of key snapshots are shown in Fig. 4b.

File Name: Supplementary_Movie10

Description: The movie of transmission imaging corresponds to upper images of Fig. 5a. The time shown in the movie is mm:ss.

File Name: Supplementary_Movie11
Description: The movie of fluorescence imaging corresponds to lower images of Fig. 5a. The time shown in the movie is mm:ss.

File Name: Supplementary_Movie12
Description: The movie of transmission imaging corresponds to upper images of Fig. 5b. The time shown in the movie is mm:ss.

File Name: Supplementary_Movie13
Description: The movie of fluorescence imaging corresponds to lower images of Fig. 5b. The time shown in the movie is mm:ss.

File Name: Supplementary_Movie14
Description: The movie of transmission imaging corresponds to Fig. 6b. Playback speed was increased by factor of three hundred sixty, namely, the movie is 30 mins long when converted to real time. The time stamps of key snapshots are shown in Fig. 6b.

File Name: Supplementary_Movie15
Description: The movie of transmission imaging corresponds to Fig. 1a before artificial fringes were removed by FFT.

References for supporting information

[1] F. Català, F. Marsà, M. Montes-Usategui, A. Farré, E. Martín-Badosa, *Sci Rep-Uk* **2017**, *7*.
